# Proteomic Analysis Reveals That P97 Inhibitors Induce Ferroptosis in Gastric Cancer AGS Cells

**DOI:** 10.3390/ph19081165

**Published:** 2026-07-26

**Authors:** Yongsheng Huang, Zhengguang Guo, Ludan Chang, Mei Huang, Lin Wang, Rongtao Lai, Chunlin Zhang

**Affiliations:** 1 Key Laboratory of Molecular Biology, School of Basic Medicine, Guizhou Medical University, Guiyang 561113, China; yongshengh83@126.com (Y.H.); 15149520025@163.com (L.C.); huangmei0505@163.com (M.H.); 2 Key Laboratory of Endemic and Ethnic Diseases, Ministry of Education, Guizhou Medical University, Guiyang 561113, China; 3 Institute of Basic Medical Sciences and School of Basic Medicine, Chinese Academy of Medical Sciences and Peking Union Medical College, Beijing 100730, China; gzg0625@sina.com (Z.G.); lin.wang@ibms.pumc.edu.cn (L.W.); 4 Department of Infectious Diseases, Ruijin Hospital, School of Medicine, Shanghai Jiaotong University, Shanghai 200020, China

**Keywords:** P97, ferroptosis, endoplasmic reticulum proteostasis

## Abstract

**Background**: P97 regulates diverse cellular processes, including proteostasis, metabolism and pluripotency. Pharmacological inhibitors of P97 protein have shown antitumor activity in various cancers. However, the downstream pathways and effectors remain poorly characterized, limiting clinical translation. **Methods**: We performed a quantitative proteomic analysis using liquid chromatography–mass spectrometry (LC–MS) to identify differentially expressed proteins (DEPs) (fold change > 1.5, *p* < 0.05) and pathway alterations in gastric cancer AGS cells following treatment with two distinct P97 inhibitors, Eeyarestatin I (EerI) and NMS873. **Results**: Proteomic analysis revealed that both inhibitors significantly perturbed multiple signaling pathways. The endoplasmic reticulum (ER) proteostasis network was markedly disrupted, evidenced by activation of the unfolded protein response (UPR) and upregulation of ER-associated degradation (ERAD) components. Pathway enrichment further indicated that cell death processes, particularly ferroptosis, were prominently affected (*p* < 0.05). Key ferroptosis regulators showed altered expression, which was validated by Western blotting. Functional assays demonstrated that P97 inhibition suppressed cell proliferation and increased the accumulation of ferroptosis-associated lipid hydroperoxides in vitro, suggesting execution of ferroptotic cell death. In vivo, using a xenograft mouse model established by subcutaneous injection of AGS cells into nude mice, P97 inhibition significantly reduced tumor growth, confirming its antitumor efficacy. **Conclusions**: This integrated proteomic and functional study delineates that pharmacological P97 inhibitors disrupt ER proteostasis and simultaneously trigger ferroptosis in gastric cancer cells. These findings identify ferroptosis as a key downstream mechanism of P97 inhibition and provide molecular mechanisms for developing P97-targeted therapies in gastric cancer.

## 1. Introduction

P97, also known as a valosin-containing protein (VCP), Cdc48 in S. cerevisiae, and TER94 in Drosophila, is an abundant AAA+ ATPase that plays an indispensable role in proteostasis and especially in the unfolding and degradation of ubiquitin-modified proteins [1,2,3,4]. For example, in mammalian cells, the accumulation of unfolded proteins in the endoplasmic reticulum (ER) activates the unfolded protein response (UPR) and elicits the ER-associated protein degradation (ERAD) [5]. In ERAD, P97, along with its cofactors NPL4 and UFD1, extract and unfold the ubiquitinated and unfolded proteins from the ER before they are degraded by the 26S proteasomes in the cytosol [6]. P97 also participates in mitochondria-associated protein degradation, as well as protein release from macromolecule complexes, membranes, ribosome and stress granules, all of which depend on the interaction between the ubiquitin moiety and P97’s adapters as in ERAD [7,8]. In the two ATPase domains (D1 and D2) of P97, ATP hydrolysis in the D2 domain fuels the unfolding and dislocation of client proteins [7,8]. Several P97 known substrates, which are degraded by the ubiquitin proteosome system, include IκB, the inhibitory protein in the NFκB pathway [9,10], hypoxia-inducible factor 1 alpha (HIF1α) [10,11], nuclear factor (erythroid-derived 2)-like 2 (NRF2) [12], TGF-β type II receptor (TβRII) [13], glutamine synthetase [14], and a large number of nuclear proteins (e.g., RNA polymerase II [15], DNA repairing proteins DDB2 [16], XPC, and Rad52 [17]). In addition to targeting specific proteins to degradation and proteostatic regulation, the loss of P97 influences a wide range of cellular processes, and many of these altered functions also result from impaired proteostasis. For example, P97 inactivity reduces tumor growth in many cancers [5,18] and the stem-like cell population in breast cancers partly through UPR [19].

Given its importance and expression in multiple cellular compartments, “omics” studies are required to comprehend the role of P97 in physiology and disease. To date, several studies used cDNA microarray and RNA sequencing to analyze the impact of P97 silencing or inhibition on cellular processes and pathways at transcriptomic level [20,21,22]. Proteomics studies so far focused on the identification of P97-interacting proteins such as the cofactors and the ubiquitin-modified proteins, whereby the protein complexes of interest are isolated by immunoprecipitation and the individual proteins are subsequently analyzed by mass spectrometry [23,24]. In these studies, the differentially expressed proteins (DEPs) were often analyzed by label-based proteomic approaches such as stable isotope labeling with amino acids in cell culture (SILAC). Recently, data-independent acquisition (DIA)-MS facilitates unbiased, proteome-wide profiling, thereby enhancing detection efficiency and sample reproducibility compared to data-dependent acquisition (DDA) [25]. Compared with the label-based approach (such as SILAC), DIA also offers simplicity and convenience to not use isotope or expensive peptide labeling tags.

In this study, we used DIA-mass spectrometry (MS) to analyze the proteomes of gastric cancer AGS cells and identified the potential downstream targets of P97 by using two pharmacological inhibitors, EerI [26] and NMS873 [27,28], which target the ER-associated P97 or globally inhibit P97 ATPase activity. EerI is a bifunctional compound with a nitrofuran ring binding to the D1 domain of P97 and an aromatic group that localizes EerI to the ER membrane [26], while NMS873 inhibits ATPase activity of D2 domain and broadly suppresses all its functions in the cell [27,28]. Because of the embryonic lethality in the P97 knockout mice and markedly reduced growth in the P97 knockdown cell lines, pharmacological inhibitors are preferred in these analyses. Our analysis detected over 5000 proteins that were differentially expressed after P97 was inhibited. Although some candidates are attributable to previously identified P97 substrates, numerous proteins and enriched pathways have not been associated with P97 in prior studies. Thus, this investigation provides interesting leads and lays the foundation for future functional characterization of undefined novel functions of P97.

## 2. Results

### 2.1. DIA-MS Detection of AGS Cell Proteomes

**Figure 1:**
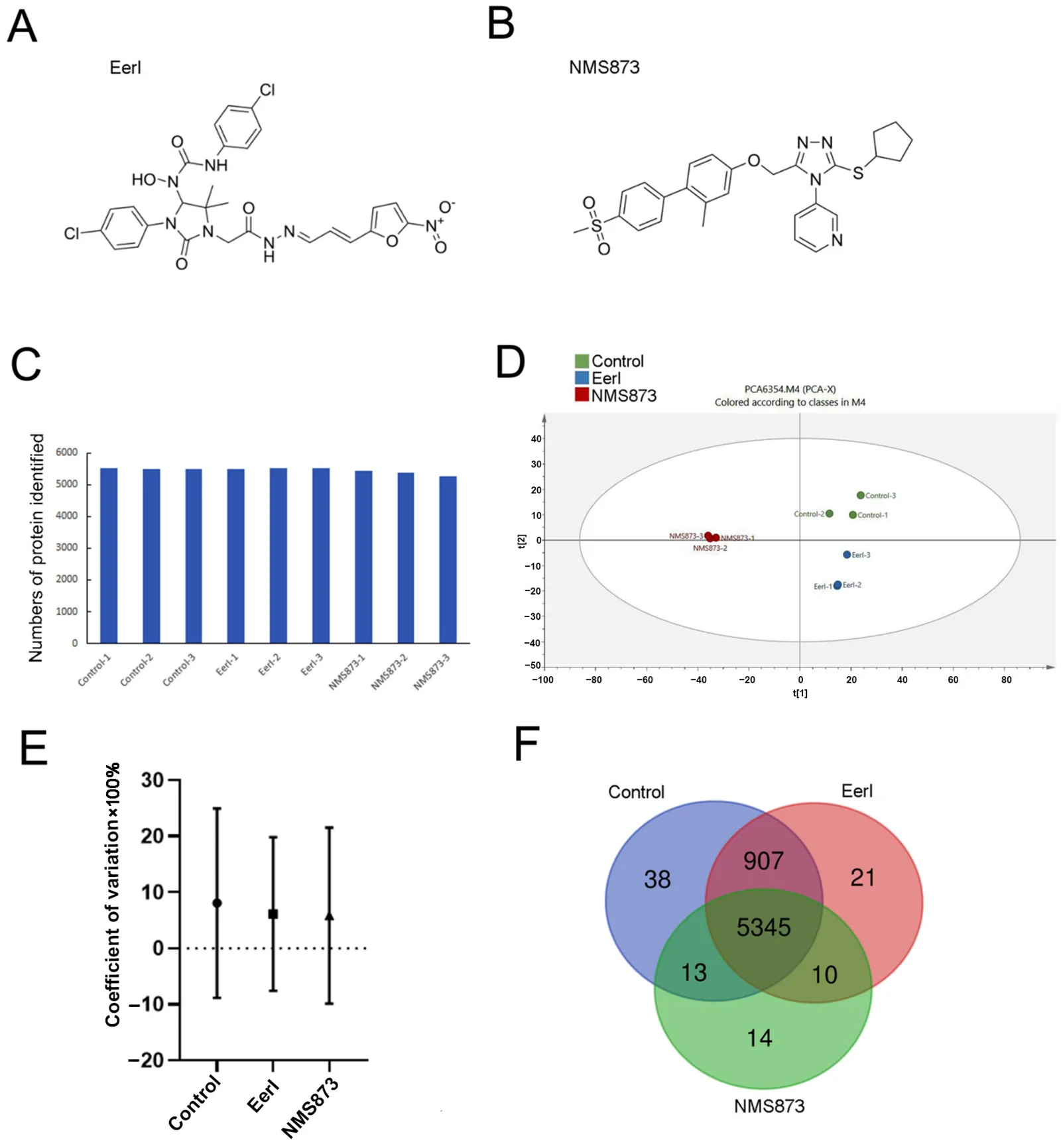
DIA-MS of all the proteomes and data quality control. (**A**,**B**) Chemical structures of EerI and NMS873. (**C**) Number of proteins detected from the DMSO, EerI and NMS873 groups by DIA-MS. (**D**) PCA plots of proteins. (**E**) The biological CV of matched proteins. (**F**) Venn diagram of the identified proteins.

The chemical structures of the known P97 inhibitors EerI and NMS873 were shown in Figure 1A,B. To characterize the potential proteins regulated by P97 in AGS cells, we treated the cells with EerI and NMS873. Dimethyl sulfoxide (DMSO) was used as a solvent control. Then, we quantitatively analyzed the proteomes of these cells using DIA-MS. A total of 6354 unique, non-redundant proteins and 60,753 peptides were identified in the DMSO, EerI and NMS873-treated AGS cells with a FDR < 1%, respectively. Figure 1C illustrates the protein identification analyses, while Supplementary Table S1 shows a full list of identified proteins. Treatment with EerI and NMS873 did not significantly affect the number of proteins detected by DIA-MS (Figure 1C). 5519 protein groups with quantitative data in more than 50% of samples in each group were selected for further analysis. The proteome difference in the DMSO-, EerI-, and NMS873-treated AGS cells was first analyzed by principal component analysis (PCA). Apparent separation trend among three conditions was observed (Figure 1D).

Data quality control was evaluated by the biological coefficient of variation (CV) of the protein abundances within the 3 groups (Figure 1E). These data demonstrate good technical reproducibility. Venn diagram showed that 5345 proteins were commonly identified in all of the 3 groups (Figure 1F). In total, 305 and 1836 significantly differentially expressed proteins (DEPs) were identified after EerI and NMS873 treatments (FC > 1.5; *p* < 0.05) (Supplementary Table S2). In particular, 185 and 120 proteins were found to be significantly up- and downregulated in the EerI-treated cells, while 1136 and 700 proteins were up- and downregulated after NMS873 treatment (Supplementary Table S2).

### 2.2. DEP-Enriched Pathways

**Figure 2:**
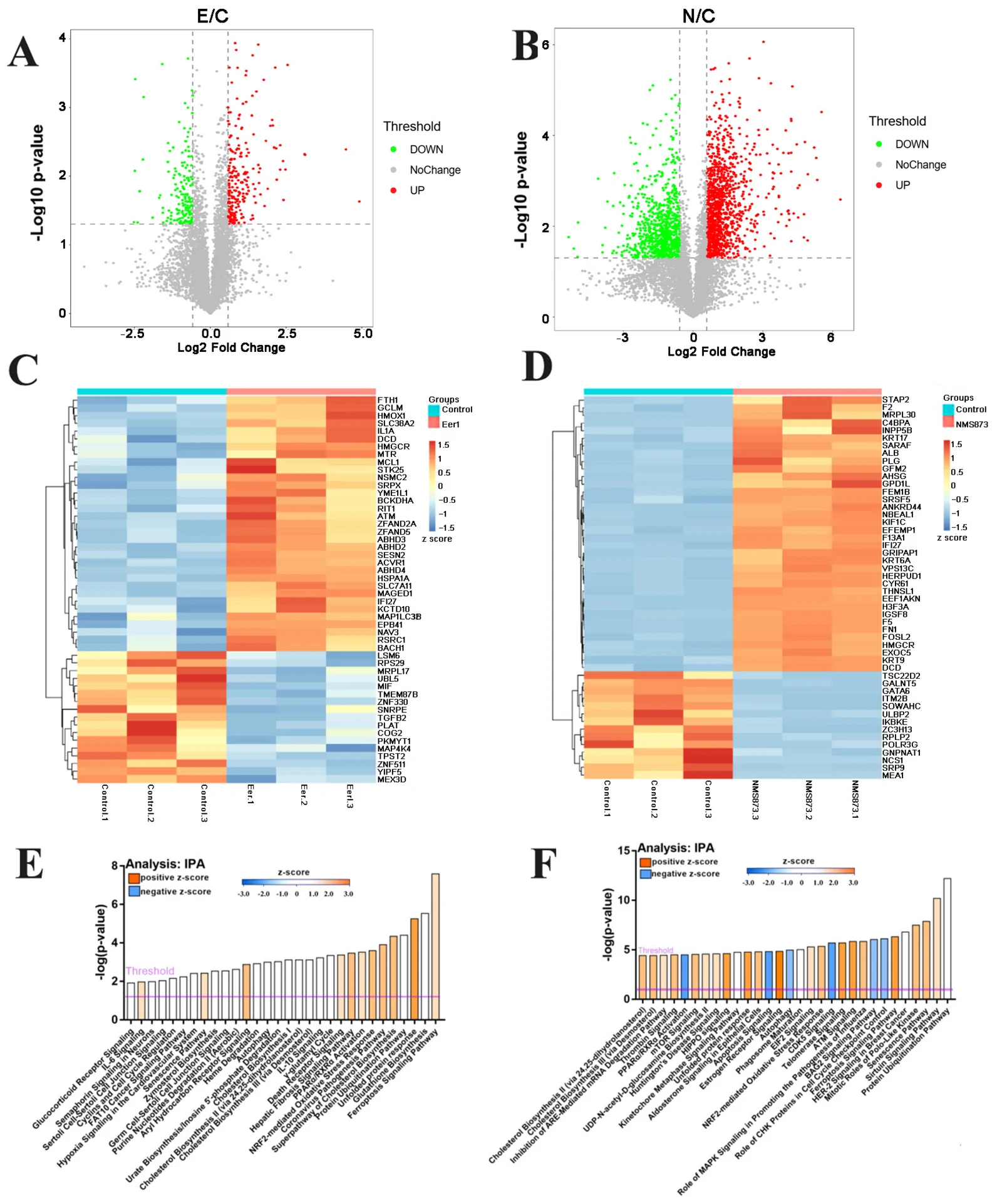
DEPs and canonical pathway analysis. (**A**,**B**) Volcano plot of DEPs found after treatment with EerI (**A**) and NMS873 (**B**) (green, down regulated, red up-regulated, grey, no change). (**C**,**D**) Heatmaps of top 50 DEPs in Eer I (**C**), and NMS873 (**D**). (**E**,**F**) Top enriched canonical pathways of DEPs in EerI (**E**) and NMS873 (**F**) from IPA.

The volcano plot demonstrates the much fewer DEPs in the EerI-treated cells, compared with the cells treated with NMS873 (Figure 2A,B). This is not surprising, as EerI only inhibits the ER-localized P97, while NMS873 inhibits the P97’s function from the entire cell. Heatmap clustering analyses of the top 50 most significant DEPs in the order of *p* values were shown in Figure 2C,D. Proteins are hierarchically clustered using Euclidean distance, generating row dendrograms that group proteins with similar expression patterns across all samples (Figure 2C,D). The *p* values for the canonical pathways were calculated from Fisher’s exact test to reject the probability of random association of molecules from the DEP dataset with the canonical pathways. Statistically significant pathways were defined as *p* < 0.05. The gene names of 305 and 1835 significant DEPs after EerI and NMS873 treatments were uploaded onto IPA online bioinformatics software (2024 Autumn version). Judged by the *p* values (*p* < 0.05), 75 and 256 pathways were found to be significantly perturbed by treatment with EerI and NMS873, respectively (Figure 2E,F).

After taking z-score (z-score ≥ 2) into consideration, we further concluded that EerI treatment activated 4 pathways, which were enriched in the upregulated DEPs and included UPR, oxidative stress response, xenobiotic metabolism general signaling, and acute phase response signaling (Supplementary Figure S1A). In contrast, treatment with NMS873 activated 30 pathways and inhibited 4 pathways based on further z-score screening (Supplementary Figure S1B). The activated pathways include UPR, nucleotide excision repair (NER), metabolism (triacylglycerol biosynthesis, fatty acid activation, fatty acid β-oxidation I, superpathway of cholesterol biosynthesis, dolichyl-diphosphooligosaccharide biosynthesis, γ-linolenate biosynthesis II, stearate biosynthesis I, CDP-diacylglycerol biosynthesis I, phosphatidylglycerol biosynthesis II), signal transduction (ATM signaling, WNT/Ca^2+^ signaling, regulation of eIF4 and p70S6K signaling, epithelial adherens junction signaling, ILK signaling, p70S6K signaling), and others (oxidative phosphorylation, endocannabinoid cancer inhibition, salvage pathways of pyrimidine ribonucleotides). The inhibited pathways (z-score ≤ –2) include sumoylation, gluconeogenesis I, and glycogen degradation II and III. The fact that EerI affected much fewer pathways is consistent with the difference in DEPs described above.

Notably, ferroptosis emerged as one of the significant perturbed pathways in the AGS cells treated with EerI (*p* value = 8.51 × 10^−7^) and NMS873 (*p* value = 1.47 × 10^−6^), despite having an insignificant activation (z-score of 0.2773 and 1.671 for EerI and NMS873, respectively (Figure 2E,F).

### 2.3. Disease and Function Analysis

**Figure 3:**
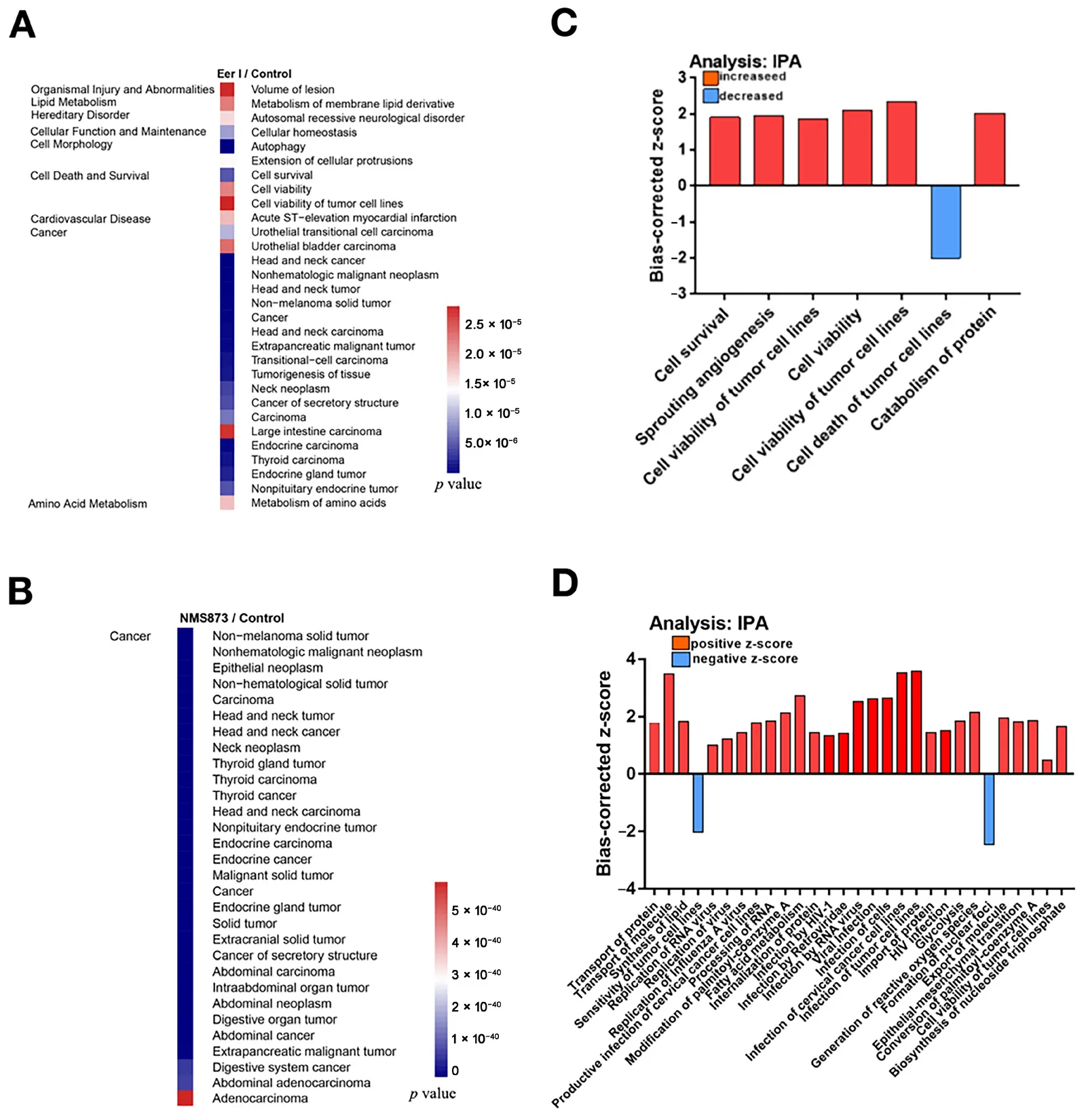
p97 inhibition-induced functional phenotypes. (**A**,**B**) Heatmaps of the top enriched functional phenotypes of DEPs upon EerI (**A**) and NMS873 (**B**) treatment. (**C**,**D**) Significant functional phenotypes following EerI (**C**) and NMS873 (**D**) exposure. Red: z-score > 0, activated; Blue: z-score < 0, inhibited.

IPA also revealed a number of downstream phenotypes (diseases and functions), which the DEPs induced by P97 inhibition might lead to. Similarly to the analysis of canonical pathways and upstream regulators, both *p* values and z-scores were taken into consideration. When only considering the *p* values, the top 30 most likely phenotypes after EerI and NMS873 treatments are illustrated in Figure 3A,B. After taking z-score into consideration, we found that EerI treatment increased 5 phenotypes and decreased 1 phenotype, while treatment with NMS873 increased 27 phenotypes and decreased 2 phenotypes (Figure 3C,D). Despite being broadly defined, a wide range of phenotypes (Figure 3C,D) including cell viability and cell death, cell invasion and differentiation, protein biogenesis and transport, metabolism, nuclear events, and stress managements were the result of P97 inhibition-induced DEPs.

### 2.4. P97 Inhibition-Induced Ferroptosis in AGS Cell

**Figure 4:**
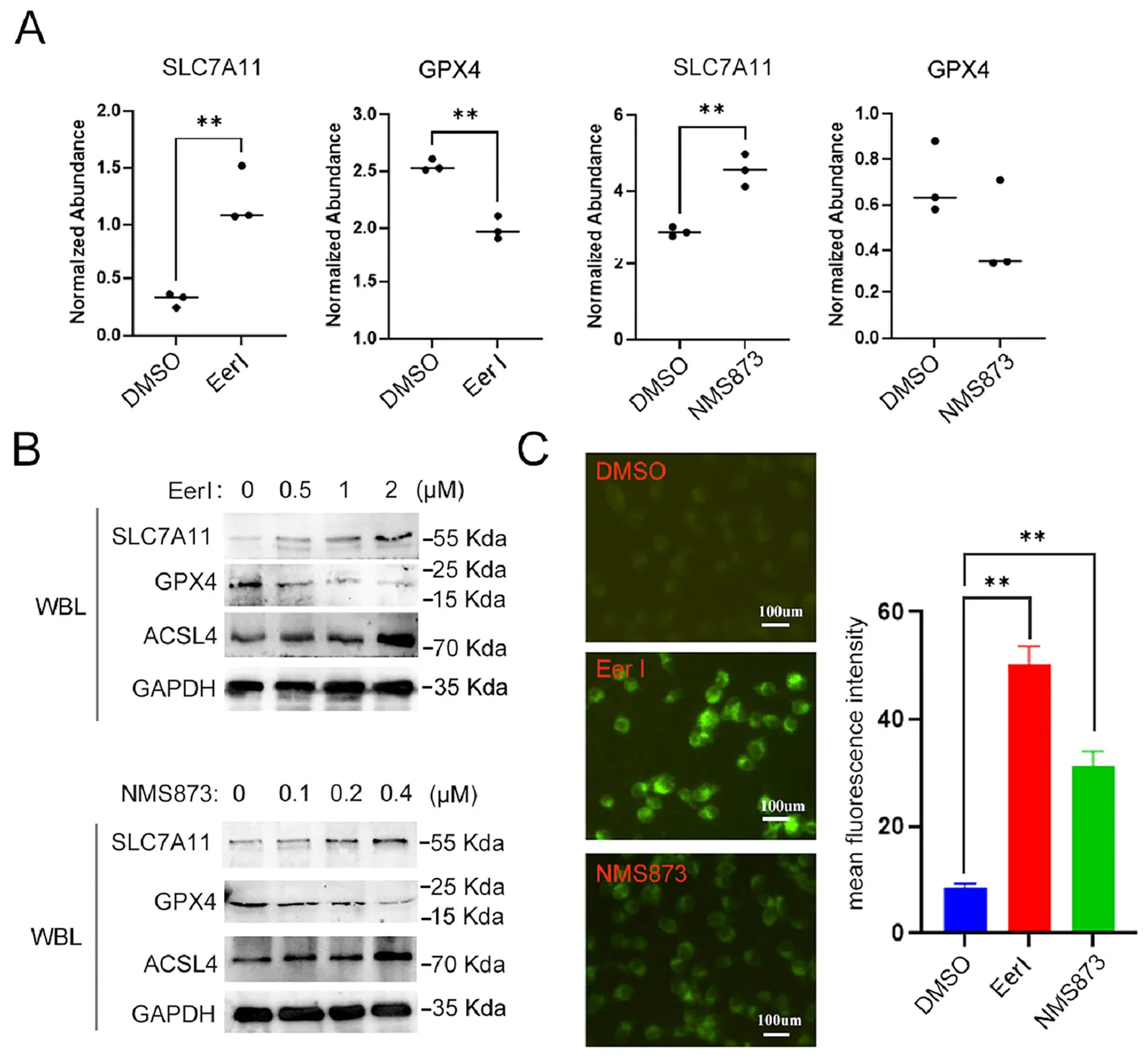
P97 inhibition triggers ferroptosis in AGS cells. (**A**) Scatter plot of the normalized abundance of the ferroptosis-related proteins in Mass spectrometry detection. ** *p* < 0.01. (**B**) Western blot detection of ferroptosis-related proteins. (**C**) Liperfluo probe staining and quantitative fluorescence intensity analysis. All data are presented as mean ± SD (*n* = 3 per group), significant difference was determined using the two-tailed Student’s *t*-test; ** *p* < 0.01 vs. DMSO group.

LC-MS proteomics demonstrated that EerI and NMS873 significantly affected ferroptosis signaling (Figure 4A). To verify whether P97 inhibition restrains AGS cell proliferation via ferroptosis, immunoblotting was performed to detect core ferroptosis regulators. P97 blockade markedly downregulated GPX4 and upregulated ACSL4 and SLC7A11 (Figure 4B). Consistently, P97 suppression triggered massive accumulation of lipid hydroperoxides, a canonical biochemical marker of ferroptosis (Figure 4C). Ferrostatin-1 rescue assays further validated this causal relationship. As shown in Supplementary Figure S2, the ferroptosis inhibitor ferrostatin-1 restored the abnormal expression of GPX4 and ACSL4 induced by EerI/NMS873 and reversed P97 inhibitor-mediated cytotoxicity in CCK-8 viability assays. These molecular and functional data together confirm that pharmacological P97 inhibition initiates ferroptosis to suppress AGS cell proliferation.

### 2.5. P97 Inhibition Reduced AGS Cell Proliferation in Xenograft Mouse Model

**Figure 5:**
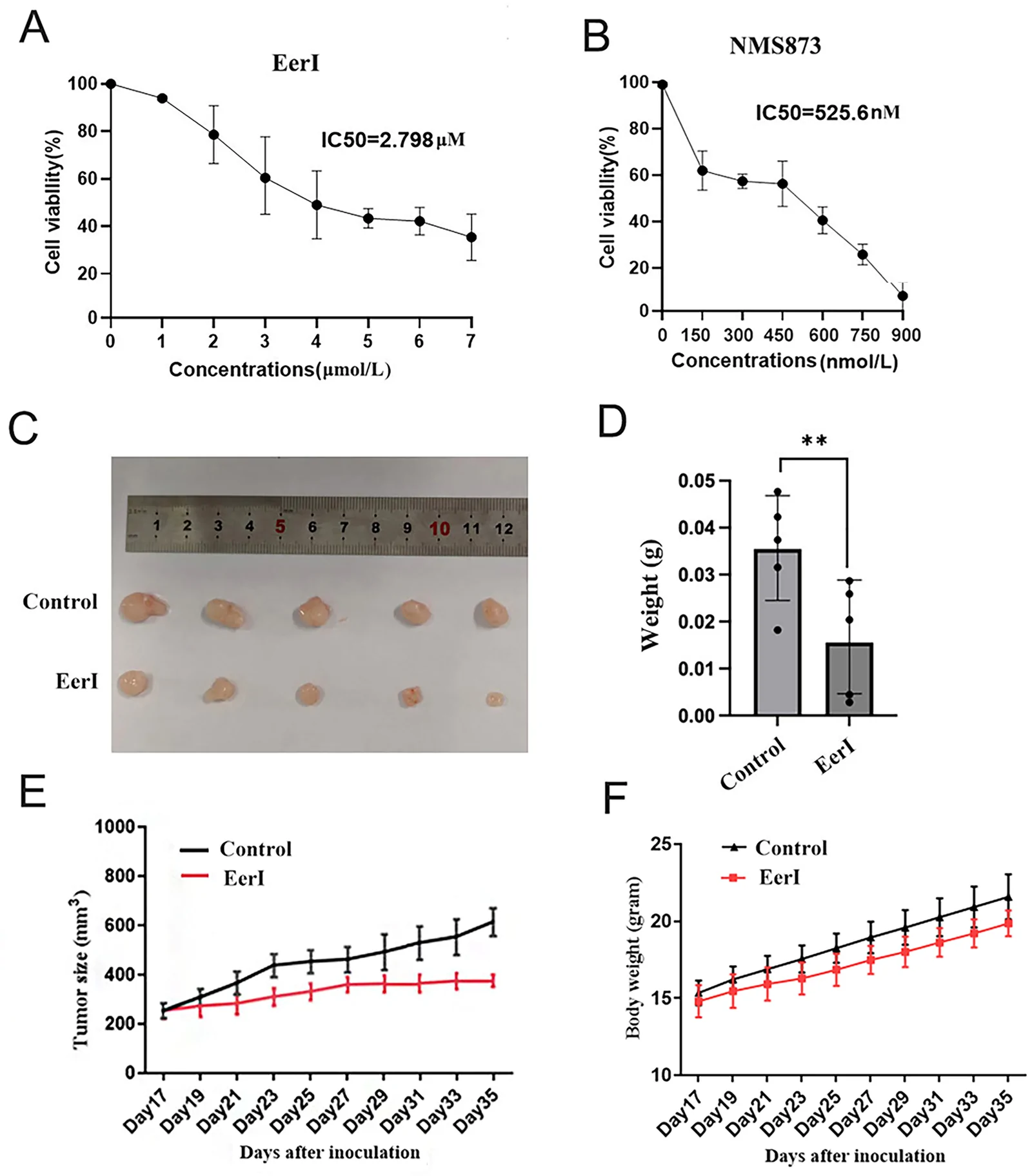
p97 inhibition suppresses AGS cell growth. (**A**,**B**) IC50 values of AGS cells treated with EerI or NMS873 for 24 h. (**C**) Representative images of subcutaneous xenograft tumors excised from nude mice receiving DMSO or EerI treatment. (**D**) Tumor weight was measured at the end of the experiment. (**E**) Tumor growth curves over the treatment period. (**F**) Body weight changes of the mice during the same period. All data are presented as mean ± SD (*n* = 5 per group). ** *p* < 0.01 vs. control.

Functionally, suppressing P97 activity with the EerI (Figure 5A) or NMS873 (Figure 5B) effectively reduced AGS cell proliferation in vitro. These findings were further validated by in vivo studies employing a xenograft mouse model. Subcutaneous injection of AGS cells into nude mice revealed that the P97 inhibitor EerI exerted a potent antitumor effect on the resulting xenograft tumors (Figure 5C–E), without significantly affecting murine body weight (Figure 5F). Histological analysis via hematoxylin and eosin (HE) staining further demonstrated that EerI led to a significant reduction in cellularity within the tumor tissues (Supplementary Figure S2). This effect is likely attributable to EerI-induced cell death, suggesting that P97 inhibition promotes tumor regression via pro-ferroptotic or other cytotoxic pathways.

## 3. Discussion

In this study, we employed a quantitative DIA-MS proteomic approach to systematically characterize the global proteomic alterations induced by pharmacological inhibition of P97/VCP in gastric cancer AGS cells. Our analysis revealed that P97 inhibition, particularly via the global ATPase inhibitor NMS873, leads to widespread changes in protein expression, impacting a diverse array of cellular pathways and biological processes. The extensive proteomic remodeling observed upon NMS873 treatment, involving over 1800 differentially expressed proteins (DEPs), underscores the central role of P97 in maintaining cellular proteostasis and homeostasis. The significantly narrower DEP profile elicited by the ER-localized inhibitor EerI aligns with its more restricted mechanism of action and previous transcriptomic observations, reinforcing the notion that P97’s functions are compartmentalized. Notably, we demonstrate that both P97 inhibitors affect ferroptosis, a regulated form of cell death driven by iron-dependent lipid peroxidation, thereby unveiling a previously underappreciated mechanism underlying their antitumor effects. Eer I specifically targets ER-localized P97 [26], whereas NMS873 globally inhibits P97 [27,28]. Despite this differential subcellular targeting, both compounds effectively trigger ferroptotic cell death. This observation implies that the ER-associated pool of P97 alone may be sufficient to govern the ferroptotic response, offering a meaningful mechanistic insight into the selective vulnerability of cancer cells. Furthermore, it raises the possibility that targeting the ER resident P97 could achieve comparable antitumor efficacy while potentially minimizing off-target toxicities associated with broad P97 inhibition.

**Figure 6:**
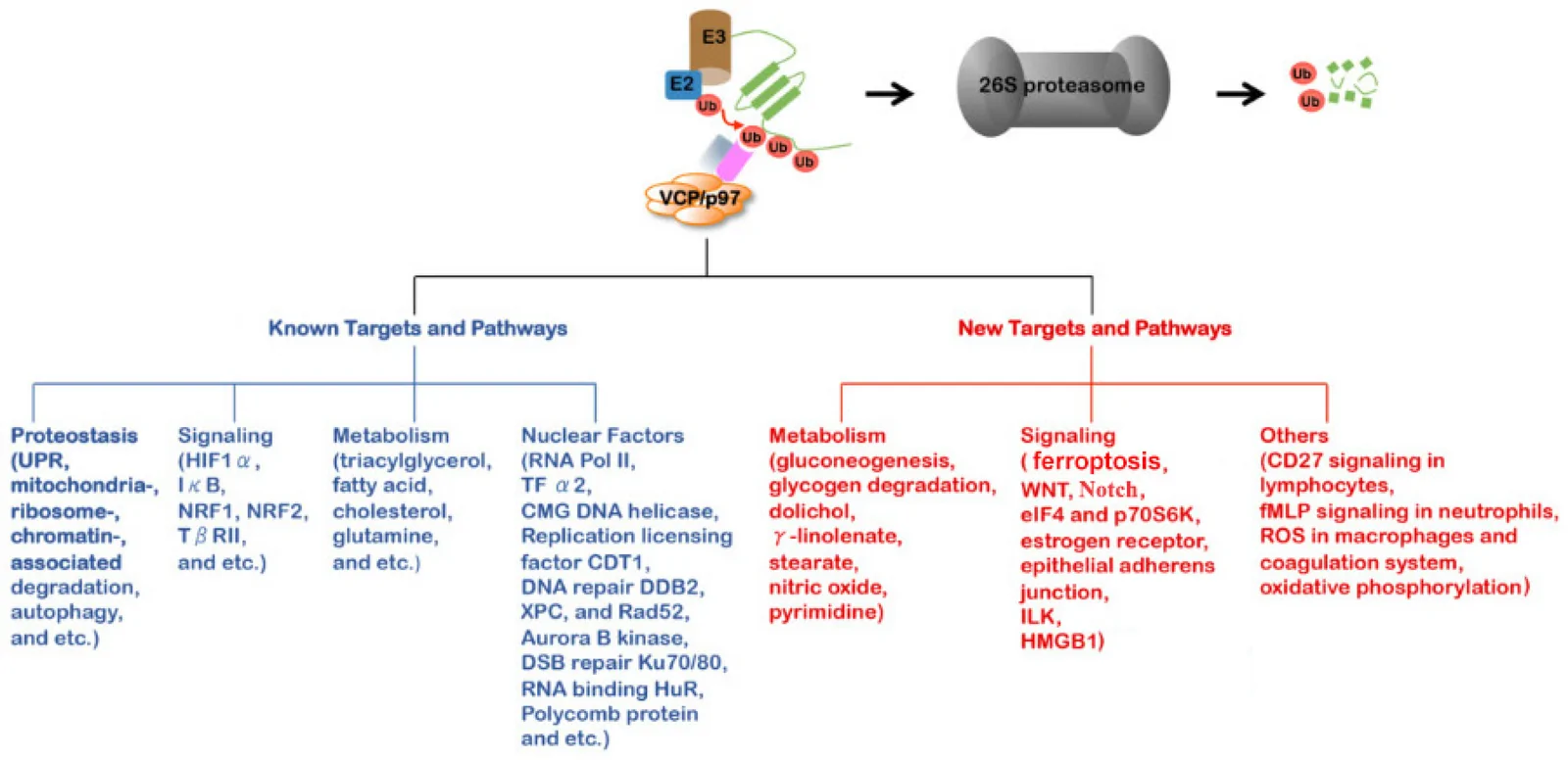
P97 regulated targets and pathways identified in this study.The blue text on the left side of the figure describes the known targets and pathways, while the red text on the right side describes newly discovered targets and pathways.

The summary of pathway enrichment analysis (Figure 6) reveals a set of P97-regulated targets and associated pathways, offering a novel mechanistic insight. This analysis delineates a mechanistic and functional landscape: the downregulation of Wnt and Notch signaling pathways, both critical for cancer cell stemness and proliferation [19], suggests one axis through which P97 inhibition impedes tumor growth. Concurrently, the upregulation of the TGFβ pathway and marked activation of the UPR and ERAD pathways highlight the severe proteostatic stress induced by P97 dysfunction, ultimately triggering cell death programs.

A key finding of our work is that pharmacological inhibition of P97 triggers ferroptosis. This conclusion is supported by multiple lines of evidence. First, proteomic analysis revealed significant enrichment of ferroptosis related pathways and key regulatory proteins. Second, functional assays confirmed increased lipid peroxidation and reduced cell proliferation, both hallmarks of ferroptotic cell death. Third, we observed that P97 inhibitors exhibit antitumor efficacy in a xenograft mouse model, underscoring the translational potential of this mechanism. Mechanistically, we propose that disrupting P97 function leads to the accumulation of ubiquitinated and misfolded proteins. This buildup aggravates endoplasmic reticulum stress and disturbs redox homeostasis. Concomitantly, our pathway analysis indicates dysregulation of lipid metabolism, particularly in triacylglycerol and fatty acid biosynthesis. Together, these perturbations create a cellular milieu that favors lethal lipid peroxidation and ultimately ferroptosis.

However, the picture is still nuanced. Ingenuity Pathway Analysis shows that while the P97 inhibitors EerI and NMS873 are significantly associated with the ferroptosis pathway with *p* values below 0.05, the z-scores do not reach the conventional threshold. This suggests that these compounds do not strongly activate or inhibit the pathway in a straightforward manner. This complexity is further reflected in the observation of increase in SLC7A11, which typically downregulated in the ferroptosis execution. Nevertheless, the expression of SLC7A11 is dynamically regulated and highly dependent on cellular context. Under conditions that favor ferroptosis, SLC7A11 is generally downregulated, whereas its upregulation occurs when cells mount a self-protective response against ferroptotic death [29,30]. This plasticity is well illustrated by the observation that p97 inhibitors can provoke an adaptive increase in SLC7A11 expression, further underscoring the intricate regulatory network that governs ferroptosis susceptibility and cautioning against over-interpretation of single marker changes. In addition, NRF2 is a known P97 substrate that is normally targeted for degradation [12]. Inhibiting P97 would therefore be expected to stabilize NRF2 and drive an antioxidant response, which would counteract, rather than promote, ferroptosis. This apparent paradox highlights the need for a more integrated view of the signaling dynamics, as the net effect may depend on the cellular context, temporal kinetics, and cross-talk between multiple pathways.

Nevertheless, our findings expand upon prior “omics” studies focused on P97. While earlier transcriptomic and interactome studies laid the groundwork [20,31], our direct proteomic quantification offers a more dynamic and functionally relevant snapshot, capturing post-transcriptional regulatory events and protein turnover changes. The identification of numerous DEPs and pathways not previously linked to P97, such as specific metabolic and immune signaling pathways, opens new avenues for research into its non-canonical functions. Moreover, recent advances in DIA-MS have enabled deeper proteomic coverage [32,33], and our study leverages this technology to provide a comprehensive landscape. However, this study has several limitations. The use of pharmacological inhibitors, while necessary due to the essential nature of P97, may have off-target effects. Although we used two distinct inhibitors to bolster specificity, genetic validation would be a valuable future step. Furthermore, the precise molecular link between P97-mediated proteostasis collapse and the initiation of ferroptosis requires further elucidation. Identifying the specific substrates or organelle stress signals are the primary drivers will be crucial.

## 4. Materials and Methods

### 4.1. Cell Culture and Reagents

Human gastric cancer cell line AGS was purchased from the Cell Resource Center of Chinese Academy of Medical Sciences and authenticated using short tandem repeat DNA profiling by Microread Genetics (Beijing, China). AGS cells were maintained in Dulbecco’s modified Eagle’s medium (DMEM) containing 10% fetal bovine serum (FBS) and 4.5 g/L glucose at 37 °C with 5% CO_2_. For proteomic analysis, each cell culture was analyzed in three replicates and the cells were lysed in a buffer [30 mM Tris-HCl (pH 8.6), 2 M thiourea, 8 M urea, and 4% *w*/*v* CHAPS] supplemented with protease inhibitors. EerI and NMS873 were purchased from MCE (MedChemExpress). HPLC-grade acetonitrile (ACN) and ammonia solution were purchased from Fisher Scientific (St. Louis, MO, USA) and Aladdin (Leonard, TX, USA). Other chemicals were purchased from Sigma-Aldrich (St. Louis, MO, USA) unless otherwise stated.

### 4.2. Sample Preparation

All samples were approximately 100 μL and the protein concentration was determined by the Bradford method. Trypsin digestion was carried out using the filter-aided sample preparation (FASP) technique. The proteins were denatured by incubation with 20 mM dithiothreitol at 95 °C for 5 min and were alkylated in 55 mM iodoacetamide in the dark for 45 min. Then, proteins were loaded into 30kD ultracentrifugation tube equilibrated with 20 mM Tris-HCl buffer, then digested with trypsin (Promega) overnight at 37 °C in the ultracentrifugation tube. The tryptic peptides were de-salted and concentrated on reverse phase C18 StageTips. The elution was dried via vacuum centrifugation.

### 4.3. LC-MS/MS Analysis

The Orbitrap Fusion Lumos Tribrid (Thermo Scientific, Bremen, Germany) coupled with an EASY-nLC 1000 was used for LC/MS analysis. The separated peptides were dissolved in 0.1% formic acid and separated on an RP C18 self-packing capillary LC column (75 μm × 150 mm, 3 μm). The eluted gradient was 5–30% buffer B2 (0.1% formic acid, 99.9% ACN; flow rate, 0.6 μL/min) and total LC duration is 60 min. The data was acquired with data-independent acquisition (DIA) under high-sensitivity mode using the following parameters. The mass spectrometer was operated in positive ionization mode. Each cycle consisted of one full MS1 scan followed by 19 fragment scans. Full scan range is from 350 to 1300 *m*/*z* and screened at 120,000 resolutions. Fragment spectra were collected at 3000 resolution and segmented 19 MS/MS scans. The maximum injection time was set to 50 ms. To evaluate the technical reproducibility of the LC–MS experiments, quality control (QC) samples were generated by pooling all the samples in equal amounts and repeatedly analyzing them throughout the entire MS process.

### 4.4. Data Analysis

The DIA raw data were imported into the Spectronaut (version 14) to calculate peptide retention time based on indexed Retention Time (iRT), and MS/MS spectra were searched by matching the retention time, *m*/*z*, to the Spectronaut peptide spectral library. For identification, the false discovery rate (FDR) was set to 0.01 on the protein and peptide levels. Proteins identified in more than 50% of the samples in each group were chosen for further analysis. Principal component analysis (PCA) was implemented using the SIMAC 15 software. Non-parameter Wilcoxon rank-sum test was performed for significance evaluation of proteins between groups. The proteins with a fold-change above 1.5 and a *p* value less than 0.05 were considered differentially expressed proteins. IPA software (2024 Autumn version)(Qiagen, Mountain View, CA, USA) was used to analyze the differentially expressed proteins, the related pathways and protein network. The Swissport accession and fold change of differential proteins were uploaded to IPA software.The proteins were mapped to the disease and function categories and canonical pathways available in IPA and were ranked by *p* values.

### 4.5. Cell Proliferation Assay

AGS cells were digested with trypsin and seeded into a 96-well plate, with three replicate wells per group. Each well was filled with 100 µL of cell suspension (approximately 5 × 10^3^ cells). After 24 h, adherent cells were treated with varying concentrations of Eeyarestatin I (0, 1.25 µM, 2.5 µM, 5 µM, and 10 µM) for another 24 h. Subsequently, 100 µL of 10% CCK8 solution was added to each well, and the plate was incubated in a humidified incubator at 37 °C with 5% CO_2_ for 1 h. Absorbance at 450 nm was measured using a microplate reader, and the half-maximal inhibitory concentration (IC_50_) of the drug was calculated using nonlinear regression analysis.

### 4.6. Detection of Intracellular Lipid Peroxide Levels

AGS cells were seeded in an 8-well chambered coverslip (3.0 × 10^4^ cells/well) and cultured overnight at 37 °C in a 5% CO_2_ incubator. After adding 2 µM EerI and incubating for an additional 24 h, the culture medium was removed, and the cells were washed once with serum-free medium. Next, 200 µL of a 1 µmol/L Liperfluo solution diluted in serum-free medium was added to each well, followed by incubation at 37 °C in a 5% CO_2_ incubator for 30 min. The solution was then removed, and the cells were washed twice with 200 µL HBSS. Cells were imaged using a confocal laser scanning microscope, and the average fluorescence intensity was analyzed.

### 4.7. Western Blot Assay

Proteins were extracted from AGS cells, and their concentrations were determined using the BCA method. The extracted proteins were mixed with loading buffer and boiled for 5 min. After adjusting the loading volume (to ensure equal protein loading across lanes), the samples were loaded for electrophoresis, followed by membrane transfer. The membrane was blocked with BSA blocking solution for 1 h, incubated with primary antibody overnight at 4 °C, washed three times with PBST, incubated with a secondary antibody for 1–2 h, washed three times with PBST, and finally developed for chemiluminescent signal detection.

### 4.8. Xenograft Study

All animal experiments were approved by the Institutional Animal Care and Use Committee of Guizhou Medical University (approve ID No.: 2400246). Four-week-old female BALB/c nude mice were subcutaneously injected with 3 × 10^6^ AGS cells in 0.1 mL PBS into the right flank. When tumors reached approximately 100 mm^3^, mice were randomly assigned to treatment groups (*n* = 5 per group), treated with DMSO, and administered intratumoral injections of EerI (10 µg/kg) every two days. Tumor volume was measured every 3 days using calipers and calculated as (length × width^2^)/2. At the end of the experiment, tumors were excised and weighed.

### 4.9. Statistical Analysis

Logarithmized ratios toward the internal standard were z-scored and clustered using Euclidean distances between averages. Significant difference was determined using the two-tailed Student’s *t*-test or the Pearson’s chi-squared (χ^2^) test, wherever appropriate. *p* value < 0.05 was considered statistically significant.

## 5. Conclusions

In summary, our integrated proteomic and functional study unveils a multifaceted mechanism through which pharmacological P97 inhibitors (EerI and NMS873) suppress gastric cancer growth. Beyond their well-established roles in disrupting canonical proteostasis networks and oncogenic signaling cascades, we uncover a previously unreported role of P97 inhibition in potentiating ferroptosis. This novel connection expands the conventional view of P97 as a mere housekeeping chaperone and positions it as a critical node integrating protein quality control with redox vulnerability in cancer cells. Importantly, our findings provide a compelling rationale for stratifying gastric tumors based on ferroptosis susceptibility markers, and strongly support future preclinical exploration of P97 inhibitors in combination with ferroptosis-sensitizing agents to achieve synergistic antitumor efficacy. Given the frequent dysregulation of P97 across diverse malignancies, this strategy may extend beyond gastric cancer to offer a broad-spectrum therapeutic opportunity, particularly for tumors characterized by high proteotoxic stress and aberrant lipid metabolism. Collectively, our work not only deepens our mechanistic understanding of P97 biology but also charts a viable path toward combinatorial regimens that exploit both proteostatic and ferroptotic vulnerabilities, potentially overcoming resistance to current therapies.

## Data Availability

The experimental material and data from this study will be available upon reasonable request.

## Supplementary Materials

The following supporting information can be downloaded at: https://www.mdpi.com/article/10.3390/ph19081165/s1. Figure S1. Significant pathways derived from DEPs; Figure S2. Ferroptosis inhibitor reverses the cytotoxicity of EerI and NMS873; Figure S3. HE staining demonstrates that P97 inhibition reduces AGS cell growth in vivo; Table S1. Full list of identified proteins by DIA-MS; Table S2. DEPs analyzed by IPA after P97 inhibition; Table S3. Upstream regulators analyzed by IPA after P97 inhibition.
